# Multimodal imaging measures predict rearrest

**DOI:** 10.3389/fnhum.2015.00425

**Published:** 2015-08-03

**Authors:** Vaughn R. Steele, Eric D. Claus, Eyal Aharoni, Gina M. Vincent, Vince D. Calhoun, Kent A. Kiehl

**Affiliations:** ^1^Mind Research Network and Lovelace Biomedical and Environmental Research Institute, AlbuquerqueNM, USA; ^2^RAND Corporation, Santa MonicaCA, USA; ^3^University of Massachusetts Medical School, WorcesterMA, USA; ^4^University of New Mexico, AlbuquerqueNM, USA; ^5^Yale University School of Medicine, New HavenCT, USA

**Keywords:** event-related potentials, functional magnetic resonance imaging, error-processing, neuroprediction, recidivism

## Abstract

Rearrest has been predicted by hemodynamic activity in the anterior cingulate cortex (ACC) during error-processing (Aharoni et al., 2013). Here, we evaluate the predictive power after adding an additional imaging modality in a subsample of 45 incarcerated males from Aharoni et al. (2013). Event-related potentials (ERPs) and hemodynamic activity were collected during a Go/NoGo response inhibition task. Neural measures of error-processing were obtained from the ACC and two ERP components, the error-related negativity (ERN/Ne) and the error positivity (Pe). Measures from the Pe and ACC differentiated individuals who were and were not subsequently rearrested. Cox regression, logistic regression, and support vector machine (SVM) neuroprediction models were calculated. Each of these models proved successful in predicting rearrest and SVM provided the strongest results. Multimodal neuroprediction SVM models with out of sample cross-validating accurately predicted rearrest (83.33%). Offenders with increased Pe amplitude and decreased ACC activation, suggesting abnormal error-processing, were at greatest risk of rearrest.

## Introduction

The revolving door of post-incarceration recidivism poses an enormous strain on society with 68% of individuals rearrested and 47% reconvicted within 3 years of release (Langan and Levin, 2002). Successfully predicting future rearrest among convicted offenders could help identify risk-factors related to reoffending. Once risk-factors have been identified, policy changes and behavioral interventions could be implemented targeting those at greatest risk. Such policies could lead to better interventions that may significantly reduce subsequent incidents of crime.

Subjective clinical predictions of future antisocial behavior (e.g., rearrest) have been shown to be highly inaccurate (Monahan, 1981). Subsequent research using empirically derived static (e.g., age, sex, criminal history) and dynamic (e.g., impulsivity, drug use, social support) risk factors have led to significant improvements in predicting future antisocial behavior (Harris et al., 1993; Douglas et al., 2002; Yang et al., 2010).

One of the strongest and most widely studied risk factors for recidivism is impulsivity or behavioral disinhibition (Harris et al., 1993; Yang et al., 2010). Impulsivity, in this context, is defined as the persistent lack of restraint and consideration of future consequences (Harris et al., 1993). Researchers have measured impulsivity in the laboratory by implementing several types of inhibition tasks (e.g., Go/NoGo, Stroop, Stop-signal, Flanker, Wisconsin Card Sorting Task, and Task-Switching: see Niendam et al., 2012 for review). A recent functional magnetic resonance imaging (fMRI) study using a Go/NoGo response inhibition task to measure cognitive control and error-processing found hemodynamic activity from the anterior cingulate cortex (ACC) measured during response errors predicted subsequent rearrest better than behavioral variables (Aharoni et al., 2013, 2014). Similarly, event-related potentials (ERPs) have been shown to be sensitive to predicting poor behavioral outcomes, including prediction of substance abuse relapse (Bauer, 1997) and failure in substance abuse treatment programs (Campanella et al., 2009; Anderson et al., 2011; Steele et al., 2014b). Here, we seek to combine ERP and fMRI measures to determine if multimodal neuroimaging measures incrementally predict rearrest with the potential of combining neuroimaging measures for more successful classification.

Studies have shown the ACC to be integral in a greater network related to cognitive control and error-processing (Carter et al., 1998; Kiehl et al., 2000; Steele et al., 2013, 2014a). Localized spatial resolution is an advantage of fMRI. In contrast, ERPs are well suited to measure rapid temporal changes that may contain important information that underlies impulse control abilities. ERP studies have shown that after a response error is made, an error-related negativity (ERN/Ne) is observed 50 ms post-error (Falkenstein et al., 1991; Gehring et al., 1993). The ERN/Ne is followed by an error positivity (Pe), peaking between 200 and 400 ms post-error (Falkenstein et al., 1991). The Pe is thought to index further error-processing, conscious evaluation of the error, response strategy adjustments, and/or affective assessment of the error (Falkenstein et al., 1991; Nieuwenhuis et al., 2001; Overbeek et al., 2005).

The ERN/Ne and Pe have been linked to cognitive control and error-processing suggesting each could be potential neural predictors of future antisocial behavior. Two competing, and somewhat overlapping, theories have been proposed to explain the underlying neural generators of cognitive control and error-processing (Holroyd and Coles, 2002; Botvinick, 2007). Though different predictions are made about specific cognitive processes and inter-neural connections, both theories implicate the ACC as a major player in cognitive control and error-processing. Neural generators of the error-related ERN/Ne and Pe elicited by an erroneous response (False Alarm) have been localized to the caudal ACC (cACC) and rostral ACC (rACC; van Veen and Carter, 2002; Edwards et al., 2012). Moreover, engagement of the ACC during conflict events in healthy adults has been shown to prospectively predict improvements in cognitive control (Kerns et al., 2004). Recent findings also suggest greater Pe amplitude (Steele et al., 2014b) and decreased ACC hemodynamic activity (Aharoni et al., 2013) elicited by False Alarms are predictors of poor future outcomes. Thus, cognitive components that engage the ACC appear to have utility in predicting future antisocial behaviors.

In the current study, neural activity was quantified using high spatial resolution fMRI and high temporal resolution ERPs and combined to index error-processes predictive of rearrest. It was expected that ERP measures indexing cognitive control and error-processing would enhance the previous findings that fMRI measures of ACC activation predict future antisocial behavior. To our knowledge, this is the first study to examine the prospective neuroprediction of both ERPs and fMRI on rearrest. Because ERN/Ne has been localized to the ACC and ACC activation has been linked to rearrest, decreased ERN/Ne amplitude also may be predictive of future antisocial behavior. Given the association between increased Pe amplitude and poor behavioral outcomes (Steele et al., 2014b), increased Pe amplitude is hypothesized to help differentiate individuals who are and are not rearrested. Multimodal neuroimaging measures of error-monitoring and post-error processes are hypothesized to be prospectively predictive of rearrest (using logistic regression and pattern classification) over 4 years, and to the imminence of rearrest (using Cox proportional hazards regression).

## Materials and Methods

### Participants

Participants were a subsample of a previously published fMRI study (Aharoni et al., 2013) who also had separate-session ERP data available. Forty-five adult male offenders ranging in age from 20 to 49 years (*M* = 32.7, SD = 7.88) with no history of significant head injury were used in these analyses. Although these samples overlap, ERP and fMRI allow for separate, unique interpretations and contributions in the identification of neural predictors of rearrest. Participants completed several psychological and behavioral assessment measures and a Go/NoGo response inhibition task prior to release from one of two New Mexico state correctional facilities. They were subsequently released and then tracked from 2007 to 2010. The average follow-up period was 23.69 months (range: 1.51–49.55 months; see Aharoni et al., 2013). Using the NIH racial and ethnic classification, 33% of the sample self-identified as White, 7% as Black/African American, 11% as American Indian, 27% as Other, 40% as Hispanic, 38% as not Hispanic, and 22% chose not to respond. Participants were informed of their right to discontinue participation at any point and that their participation was in no way associated with their status at the facility or their parole status, and there were no direct institutional benefits. They were paid $1 per hour, a rate commensurate with standard pay for work assignments at their facility. Participants provided written informed consent in protocols approved by the institutional review board of the University of New Mexico.

### Assessments

Rearrest data, including arrest date and charge, were obtained by a professional criminal background check service (SSC, Inc.), which conducted national, state, and county criminal searches following each participant’s release date. Approximately 53% of the sample was rearrested between their release date (ranging from 2007 to 2010) and the follow-up date of September, 2011. The outcome variable for Cox regression analyses (described below) was the number of days between release from incarceration and the follow-up date or the subject’s rearrest date, whichever came first. Offense type was classified into one of 27 common felony categories by 10 trained raters. In line with previous literature (Corrado et al., 2004), offenses were classified as violent or non-violent offenses and minor parole and probation violations were not included. Because very few participants were rearrested for violent offenses (*N* = 8; 17.8%), analyses were carried out on reoffense status rather than violent offense status. Of the 45 individuals who had both ERP and fMRI data, 24 were rearrested during the follow-up period.

Data from several additional potential risk factors were obtained to examine the incremental predictive validity of the ERP and fMRI measures. Behavioral indices of disinhibition included scores from the Hare Psychopathy Checklist-Revised (PCL-R; Hare, 2003), behavioral False Alarm rates calculated separately for ERP and fMRI (defined as the proportion of observed False Alarms out of total NoGo trials), age at release, and lifetime prevalence of drug and alcohol abuse/dependence (assessed using the Structured Clinical Interview for the Diagnostic and statistical manual-IV: research version; SCID I; First et al., 1997). Abuse and dependence were defined by diagnostic scores of 2 and 3, respectively. An average drug abuse/dependence measures was calculated with an average score from the following drug classes: sedatives (11% met for dependence), cannabis (53% met for dependence), stimulants (53% met for dependence), opioids (22% met for dependence), cocaine (62% met for dependence), and hallucinogens (11% met for dependence). Over half (51%) of participants met SCID criteria for alcohol dependence. All of these variables are known predictors of antisocial behavior in offender populations or are correlated with ACC activity (Kerns et al., 2004; Monahan, 2008; Simmons et al., 2008).

### Stimuli and Task

Behavioral impulsivity was measured using the Go/NoGo response inhibition task, a widely used procedure that requires participants to inhibit pre-potent motor responses. The Go/NoGo task (Kiehl et al., 2000) consisted of two experimental runs, each comprising 245 visual stimuli. The stimuli were presented to participants using the computer-controlled visual and auditory presentation software package, Presentation^®^ (www.neurobs.com). Each stimulus appeared for 250 ms in white text within a continuously displayed rectangular fixation box. Participants were instructed to respond as “quickly and accurately as possible” with their right index finger every time the target (“Go”) stimulus (a white “X”) appeared, and to withhold a response when the distracter “No/Go” stimuli (a white “K”) appeared. Targets appeared with higher frequency (84%; 412 trials; 206 for each run) to establish a strong stimulus-response mapping on “Go” trials. Two K’s were never presented sequentially. The stimuli were approximately 3 × 5 visual degrees on a black background. The interstimulus interval was jittered (1–3 s stimulus onset asynchrony; averaging 1.5 s). Prior to recording, each participant performed a block of 10 practice trials to ensure that the instructions were clearly understood. The standard onset asynchrony (SOA) between Go stimuli varied pseudo-randomly between 1000, 2000 and 3000 ms, subject to the constraint that three Go stimuli were presented within each consecutive 6 s period. The NoGo stimuli were interspersed among the Go stimuli in a pseudorandom manner subject to three constraints: the minimum SOA between a Go and NoGo stimulus was 1000 ms; the SOA between successive NoGo stimuli was in the range 8–14 s. The same task procedures were used in both ERP and fMRI data collection.

### Data Acquisition

Magnetic resonance image acquisition parameters were discussed previously (Aharoni et al., 2013) and are only briefly outlined here. Images were collected with a mobile Siemens 1.5T Avanto with advanced SQ gradients (max slew rate 200 T/m/s 346 T/m/s vector summation, rise time 200 us) equipped with a 12 element head coil. The EPI gradient-echo pulse sequence (TR/TE 2000/39 ms, flip angle 75°, FOV 24 cm× 24 cm, 64 × 64 matrix, 3.4 mm × 3.4 mm in plane resolution, 5 mm slice thickness, 30 slices) effectively covers the entire brain (150 mm) in 2000 ms. Head motion was limited using padding and restraint.

Electrophysiological data were collected in a separate session that the fMRI data collection using two Windows-compatible computers and a 64-channel BioSemi ActiveTwo amplifier. The first computer used Presentation^®^ software (www.neurobs.com) to deliver the stimuli, accept responses, and send digital triggers to the other computer indicating when a stimulus or response occurred. The second computer acquired physiological data using BioSemi software and amplifier. All signals collected with this BioSemi system were low-pass filtered using a fifth order sinc filter with a half-power cutoff of 204.8 Hz then digitized at 1024 Hz during data collection. Electroencephalography (EEG) activity was recorded using sintered Ag–AgCl active electrodes placed in accordance with the 10–20 International System (Jasper, 1958). The participant’s nose was used as the reference. Six electrodes were placed on the participants face to measure electro-oculogram. These electrodes were placed above, below, and on the canthus of each eye. All offsets were kept below 10 kΩ.

### Data Reduction

Functional images were reconstructed oﬄine at 16-bit resolution and manually reoriented to approximately the anterior commissure/posterior commissure (AC/PC) plane. Functional images were spatially normalized to the Montreal Neurological Institute (MNI) template via a nine-parameter affine transformation using smooth basis functions to account for non-linear differences, and spatially smoothed (8 mm full-width half maximum) in SPM5. High frequency noise was removed using a low-pass filter (cutoff – 128 s). Response types (Hits and False Alarms) were modeled as separate events. Event-related responses were modeled using a synthetic hemodynamic response function composed of two gamma functions. The first gamma function modeled the hemodynamic response using a peak latency of 6 s. A term proportional to the derivative of this gamma function was included to allow for small variations in peak latency. The second gamma function and associated derivative was used to model the small “overshoot” of the hemodynamic response on recovery. A latency variation amplitude-correction method was used to provide a more accurate estimate of hemodynamic response for each condition that controlled for differences between slices in timing and variation across regions in the latency of the hemodynamic response (Calhoun et al., 2004).

**FIGURE 1:**
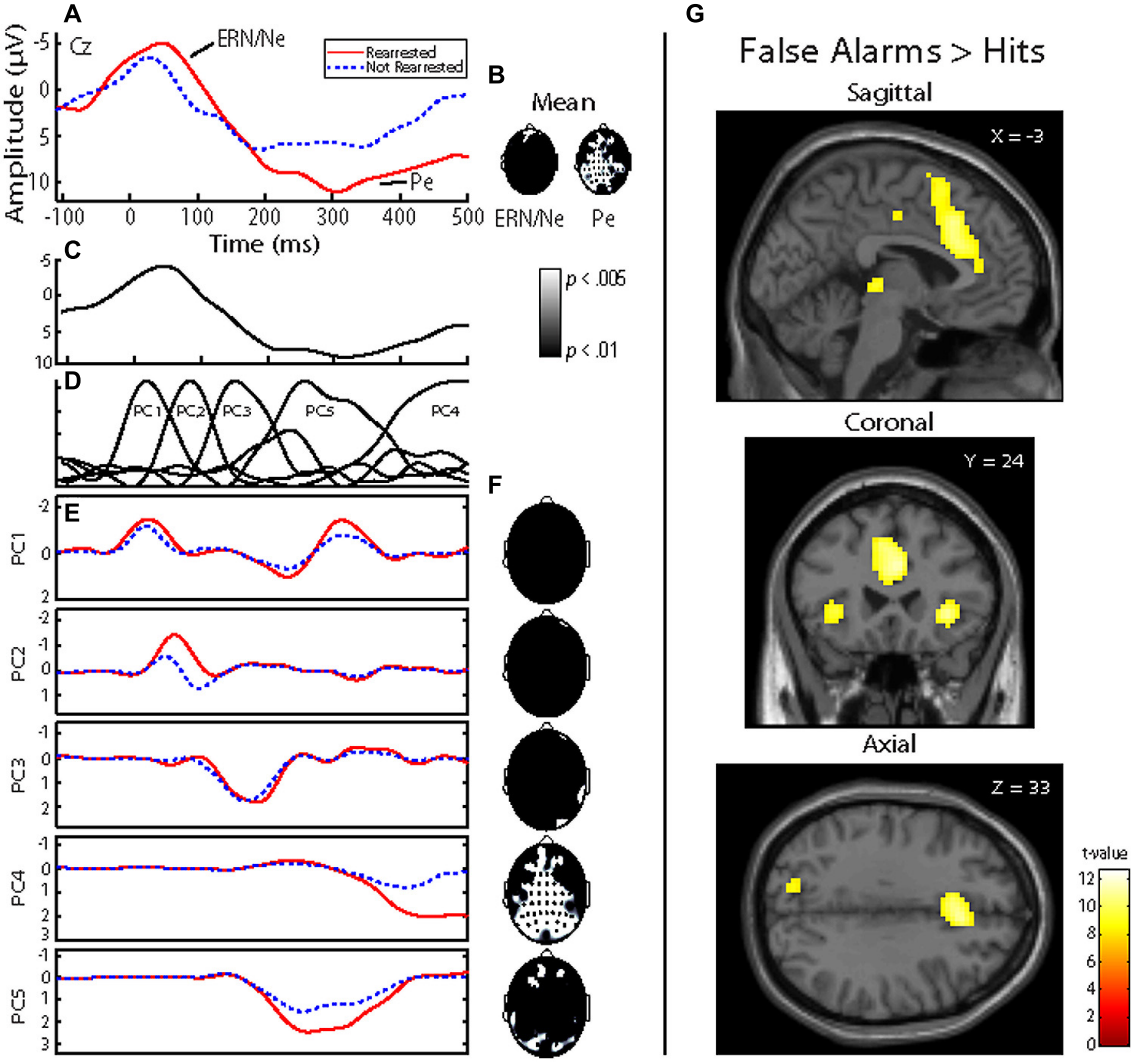
**Event-related potential (ERP) and functional magnetic resonance imaging (fMRI) analysis of False Alarms. (A)** Representative ERP waveform plotted at Cz for each group. Negative is plotted up. Individuals who were rearrested (solid red line) and not rearrested (dashed blue line) are plotted. ERP components of interest (ERN/Ne and Pe) are identified. **(B)** Topographical statistical difference (black and white) maps are plotted for each component window highlighting individuals who were rearrested exhibited increased Pe amplitude. Groups did not exhibit different ERN/Ne amplitudes. **(C)** Grand average waveform plotted at Cz. **(D)** A five-component principal component solution accounting for 93.59% of the variance. **(E)** Group average waveforms for individuals who were rearrested (solid red line) and not rearrested (dashed blue line are plotted ad Cz. **(F)** Topographical statistical (black and white) maps are plotted for each principal component highlighting individuals who were rearrested exhibited increased PC4 amplitude. **(G)** Hemodynamic response differences between False Alarm and Hits contrast. Sagittal, coronal, and axial slices are plotted centered at the ACC coordinate of interest, x = -3, y = 24, z = 33. Family wise error *p* < 0.00001 was implemented to account for multiple comparisons.

Functional images were computed for each participant that represented hemodynamic responses associated with False Alarms and Hits. General linear models included regressors to model motion (six parameters). Activation difference between False Alarms and Hits was extracted from a 14 mm radius sphere centered in the ACC (-3, 24, 33; **Figure 1**). This ACC coordinate has been previously identified (Kiehl et al., 2000; Steele et al., 2014a) and was the same coordinate used to predict future rearrest (Aharoni et al., 2013).

Electroencephalography data pre-processing steps included down sampling to 512 Hz, bad channel detection and replacement, epoching, and eye-blink removal. Bad channels were identified as having activity four standard deviations away from the mean of all electrodes placed on the scalp. These channels were replaced using the mean of surrounding scalp electrodes. ERP epochs were defined in relation to responses, from 1000 ms pre- to 2000 ms post-response. The epoched data were eye-blink corrected using an independent component analysis (ICA) technique. The ICA utility in the EEGLab software (Delorme and Makeig, 2004) was used to derive components then, using an in-house template matching algorithm (Jung et al., 2000), blink components were identified and removed from the data. Individual subject ICA decompositions where no eye-blinks were identified and removed were visually inspected to identify eye-blink components which, when present, were then removed.

The ERN/Ne component window was defined as the negative deflection that occurred between -100 ms to 115 ms relative to a False Alarm. The Pe component window was defined as the positive deflection that occurred between 75 and 500 ms relative to a False Alarm (**Figure 1**). These ERP components were defined to best fit these data and were baseline corrected using a -200 ms to -110 ms window, relative to a False Alarm. Within each trial, individual electrodes in which activity exceeded ± 100 μV were omitted from analyses. Applying these criteria, 15.12% of electrode trials were excluded. ERN/Ne amplitude was maximal at FCz and Pe amplitude was maximal at CPz. Therefore, these electrodes were selected as most representative of ERN/Ne and Pe related activations and used in subsequent analyses. An additional data reduction method, principal component analysis (PCA; Chapman and McCarry, 1995), was also performed on False Alarm trials. This method is optimal for ERP data analysis because classic windowed component time-domain (TD) measures of ERP are inadequate at separating the inherently overlapping ERP components (Dien et al., 2007). We have previously used this method (Steele et al., 2014b) and highlight PCA measures are more sensitive in predicting outcomes in similar models as presented here. A five-component solution was extracted (**Figure 1**) which accounted for 93.59% of the variance.

### Data Analysis

Three analytical approaches were used to prospectively predict rearrest: (1) Cox proportional hazards regressions were used to predict time to rearrest. Cox regression takes ‘time at risk’ into account by using time to rearrest as the outcome variable, calculated as the number of days between release from incarceration and the rearrest date, or the follow-up date (September 2011) for those who were not rearrested. Those who were not rearrested are included in the analyses and considered to be ‘censored’ cases, meaning they potentially could still reoffend, accounting for variable lengths of follow-up. Reliability of the Cox regressions was assessed by using bootstrapping with 9,999 iterations; (2) Logistic regressions were used to test linear combinations of variables in identifying the occurrence of a rearrest, without taking time at risk into account. A binary outcome variable of rearrested or not rearrested between release from incarceration and follow-up date was used. Measures of overall performance, sensitivity specificity, and area under the curve were calculated for each logistic model. Participants were classified using leave-one-out cross-validation; (3) Support vector machines (SVMs; i.e., pattern classifiers) were used to test non-linear combinations of variables in identifying the occurrence of rearrest. A binary outcome variable of rearrested or not rearrested between release from incarceration and follow-up date was used. Measures of overall performance, sensitivity, and specificity were calculated for each SVM model. SVMs are especially beneficial when data classes are heterogeneous with few training samples (Melgani and Bruzzone, 2004). This binary classifier finds a hyperplane that maximizes the margin between two classes. Participants were classified using two nested leave-one-out cross-validations. In each iteration, one participant is selected as the testing sample and the rest as training samples (first leave-one-out). To select the best parameter for the SVM classifier, a grid search was performed over parameters C and σ. C is the value of the box constraint for the soft margin and σ is the scaling factor of the rbf kernel. Values for C were in this set {C = 2^∧^ -9, 2^∧^ -8.5…2^∧^ -4} and values for σ were in this set {σ = 2^∧^-2, 2^∧^-1.5…2^∧^4}. The classification rate was measured for each parameter set using another leave-one-out validation inside the training set. The best C and σ were identified by the model that produced the greatest combination of sensitivity and specificity. After selecting the best parameter, the left out testing sample was classified. Matlab version 7.12.0 (R2011a) was used to implement the svmtrain and svmclassify functions and a Gaussian radial basis function (rbf) kernel to develop these classification models. Within each model, the variables were z-scored to standardize across the variable set. This procedure (using two nested leave-one-out) avoids any use of training data in model selection or model training, which is crucial in any classification problem. This method has been used successfully with other datasets in our laboratory (Cope et al., 2014; Steele et al., 2014b).

Four models were calculated for each of the analytical approaches used to prospectively predict rearrest described above: Model 1 included ERP measures (either TD or principal component measures of ERN/Ne and Pe), and all covariates (age at release, PCL-R Factor 1, PCL-R Factor 2, drug abuse/dependence, and alcohol abuse/dependence measures; **Table 1**); Model 2 included ACC activation and all covariates; Model 3 included ERP measures, ACC activation, and all covariates; Model 4 included ERP measures and ACC activation. These four models were designed to best identify the unique and overlapping contributions of the neural measures of error-processing in predicting rearrest. Using three analytical approaches with four models allowed for analysis of both a binary (i.e., were or were not rearrested) and a continuous (i.e., time to rearrest) outcome. The outcomes of these analyses should ultimately influence future attempts at prospectively predicting when and if rearrest is likely.

**Table 1:** Descriptive statistics and independent samples *t*-tests for variables used as covariates.

	All participants (*N* = 45)			Rearrested group (*N* = 24)			Not-rearrested group (*N* = 21)		
Variable	*N*	Mean	SD	*N*	Mean	SD	*N*	Mean	SD	*t*	*df*	*p*
Age at release	45	33.69	8.08	24	31.58	7.32	21	36.10	8.40	1.93	43	0.061
PCL-R-F1	42	7.64	3.26	22	7.55	3.49	20	7.74	3.07	0.18	40	0.853
PCL-R-F2	42	14.31	3.79	22	15.17	3.39	20	13.36	4.05	1.69	40	0.099
Drug abuse/dependence (Lifetime)	44	2.89	1.30	23	2.91	1.28	21	2.86	1.35	-0.14	42	0.888
Alcohol abuse/dependence (Lifetime)	44	2.27	0.85	23	2.22	0.85	21	2.33	0.86	0.45	42	0.655
NoGo accuracy (ERP)	45	77%	0.13	24	74%	0.15	21	80%	0.10	1.58	43	0.123
NoGo accuracy (fMRI)	45	74%	0.14	24	70%	0.15	21	78%	0.11	1.86	43	0.069

*All participants (*N* = 45) either subsequently rearrested or not rearrested. Individuals in the rearrested group (*N* = 24) include adult male offenders who were rearrested within the follow-up period. Individuals in the not rearrested group (*N* = 21) include adult male offenders who were not rearrested within the follow-up period. PCL-R-F1 and PCL-R-F2 are factor scores derived from the Hare Psychopathy Checklist-Revised (PCL-R; Hare, 2003). Factor 1 is a measure of 13 interpersonal and affective traits. Factor 2 is a measure of antisocial and impulsive traits.*

## Results

Behavioral, fMRI measures, ERP component windowed TD, and PCA measures for False Alarms were included, along with the additional covariates described above, in Cox proportional hazards regressions, logistic regressions, and SVM models. Zero-order effects of neural measures (ERP and fMRI) of error-monitoring and post-error processing were initially calculated for each analysis model before adding all covariates. Because the ERP TD and PCA measures are each derived from the same neural signal, separate TD and PCA models were calculated. To identify which principal component accounts for which ERP windowed TD measure (ERN/Ne and Pe), linear regressions were performed using the five principal components predicting TD components. ERP models included either TD or PCA measure or the ACC activity measured during fMRI data collection. Effects that did not reach statistical trend (*p* > 0.10) are not reported.

Consistent with prior studies, response times measured in both ERP and fMRI for False Alarms (ERP: *M* = 333 ms, SD = 46 ms; fMRI: *M* = 359 ms, SD = 44 ms) were faster than for Hits (ERP: *M* = 659, SD = 55 ms; fMRI: *M* = 599 ms, SD = 42 ms), *t*(44) = 25.01, *p* < 0.001, *t*(44) = 64.44, *p* < 0.001, respectively. Participants also were more accurate to Go (ERP: 98%; fMRI: 98%) than NoGo (ERP: 77%; fMRI: 74%) trials, *t*(44) = 9.66, *p* < 0.001, *t*(44) = 11.60, *p* < 0.001, respectively.

About half of the sample (*N* = 24) was rearrested during the 4-years follow-up period. The group that was rearrested was marginally younger, *t*(43) = 1.93, *p* = 0.061, scored higher on PCL-R Factor 2, *t*(40) = 1.69, *p* = 0.099, and made more False Alarms in the fMRI task, *t*(43) = 1.86, *p* = 0.069, than the group that was not rearrested. The groups did not differ on the other behavioral measures or other covariates used in analyses below (see **Table 1**). The outcome variable for Cox regression (days to rearrest or days to follow-up date for those who were not rearrested), was shorter for the rearrested group (*M* = 11.89 months, SD = 10.77 months, range: 1.51–11.89 months) than the non-rearrested group (*M* = 34.02 months, SD = 9.19 months, range: 10.38–49.55 months), *t*(43) = 7.44, *p* < 0.001. This outcome variable was marginally correlated with ACC activation, *r* = 0.256, *p* = 0.090, but not other variables used in the models below (*r’s* < 0.23).

### Regressions

To identify which principal component best describes each ERP component, linear regressions were computed predicting mean TD (i.e., ERN/Ne and Pe) amplitudes with the five principal components (see **Figure 1** for TD and PCA representations of these data). Mean ERN/Ne amplitude was predicted by principal component 1 (PC1), *p* = 0.006, and principal component 4 (PC4) was a marginal predictor, *p* = 0.091 (**Table 2**). Mean Pe amplitude was predicted by PC4, *p* = 0.001 (**Table 2**). Therefore, separate analyses were carried out using TD measures (ERN/Ne and Pe) and principal component measures (PC1 and PC4) as neural measures of error-monitoring (ERN/Ne and PC1) and post-error processing (Pe and PC4).

**Table 2:** Summary of linear regression analysis of principal components predicting windowed time-domain (TD) components (*N* = 45).

	Predictors	*B*	SE *B*	β
**Regression 1**				
DV ERN/Ne mean				
	PC1 mean	12.806	4.384	0.538^∗^
	PC2 mean	-2.547	3.612	-0.219
	PC3 mean	-0.423	2.904	-0.041
	PC4 mean	4.458	2.569	0.306^∧^
	PC5 mean	1.595	2.815	0.125
**Regression 2**				
DV Pe mean				
	PC1 mean	7.442	6.166	0.136
	PC2 mean	4.075	3.630	0.218
	PC3 mean	4.326	2.791	0.291
	PC4 mean	10.300	2.846	0.474^∗^
	PC5 mean	-2.684	3.725	-0.103

*Regression 1: *R^*2*^*.30 *R.*55 (*p* = 0.013); Regression 2: *R^*2*^*.65 *R.*81 (*p* < 0.001). ERN/Ne is the mean amplitude of the error-related negativity (ERN/Ne) ERP component measured at FCz for responses to NoGo stimuli; Pe is the mean amplitude of the error-related positivity (Pe) ERP component measured at CPz for responses to NoGo stimuli. Principal components used to predict TD components were measured at the corresponding electrode for responses to NoGo stimuli. ^∧^*p* < 0.10, ^∗^*p* < 0.05.*

### Prediction Models

Cox regressions were computed to identify variables useful in predicting time to rearrest. Zero-order effects calculated for Cox regressions predicting rearrest with ERP TD, ERP PCA, and ACC activation measures were first computed. Only Pe and PC4, not ERN/Ne, PC1 or ACC activation, were significant predictors of rearrest (**Table 3**). When additional covariates were added to the models, Pe and PC4 remained significant and age at release, PCL-R Factor 1, PCL-R Factor 2, and ACC activation were significant or marginally significant (**Tables 4** and **5**). While accounting for other covariates and ACC activation, PC4 was the greatest predictor of time to rearrest in that for every unit increase in amplitude, the probability of rearrest increased 8.13 times, *p* = 0.004 (**Table 5B**). Interestingly, PC4 remained the only unique predictor when only ERP and fMRI measures were included in the model, *p* = 0.039 (**Table 5D**). Bootstrapped Cox regression results suggest these models to be relatively stable.

**Table 3:** Zero-order Cox and logistic regressions with ERN/Ne, Pe, PC1, PC4, and ACC activation predicting rearrest (*N* = 45).

(A)							
Model	-2 Log likelihood	Overall			Change from previous		
		χ^2^	df	*p*-value	Δχ^2^	df	*p*-value
*(A) ERN/Ne*	160.35	0.28	1	0.597	0.29	1	0.588
*(B) Pe*	155.81	4.88	1	0.027^∗^	4.83	1	0.028^∗^
*(C) PC1*	159.94	0.67	1	0.413	0.71	1	0.400
*(D) PC4*	154.06	6.55	1	0.010^∗^	6.59	1	0.010^∗^
*(E) ACC*	158.75	1.97	1	0.161	1.90	1	0.169

*Omnibus test of Cox regression Model with Chi-square statistics (χ^*2*^) showing the zero-order effect of mean ERN/Ne (A), Pe (B), PC1 (C), PC4(D), and ACC (E) activity on months to rearrest.*								

**(B)**								
**Model**	***B***	**SE *(B)***	***p*-value**	**exp[B]**	**CI (95%) for exp[B]**	-**2 Log likelihood**	**Cox and Snell *R^2^***	**Nagelkerke *R*^2^**

*(A) ERN/Ne*	0.07	0.07	0.363	1.07	0.93–1.23	59.87 (0.32)	0.02 (<0.001)	0.02 (0.001)
*(B) Pe*	0.15	0.06	0.022^∗^	1.16	1.02–1.31	53.84 (0.95)	0.14 (0.003)	0.19 (0.005)
*(C) PC1*	-0.62	1.68	0.702	0.57	0.02–16.09	60.61 (0.18)	0.002 (0.002)	0.003 (0.003)
*(D) PC4*	4.22	1.61	0.009^∗^	82.45	3.05–2712.0	50.77 (1.30)	0.20 (0.006)	0.27 (0.008)
*(E) ACC*	-0.54	0.38	0.157	0.58	0.28–1.22	58.56 (0.50)	0.05 (0.001)	0.06 (0.002)

*Omnibus test of logistic regression, with cross-validation, model showing the zero-order effect examining the predictive effect of ERN/Ne (A), Pe (B), PC1 (C), PC4(D), and ACC (E) on rearrest. -2 Log likelihood, Cox and Snell, and Nagelkerke are presented for each model with standard deviations presented in parentheses. (A) Model χ^2^(1) = 0.86 (SD = 0.04), *p* = 0.354. Classification of rearrested: 70.83% (17 of 24); not rearrested: 28.57% (6 of 21); overall 51.11% (23 of 45). AUC = 0.497. (B) Model χ^2^(1) = 6.99 (SD = 0.19), *p* = 0.008. Classification of rearrested: 66.67% (16 of 24); not rearrested: 57.14% (12 of 21); overall 62.22% (28 of 45). AUC = 0.619. (C) Model χ^2^(1) = 0.09, *p* = 0.757. Classification of rearrested: 79.17% (19 of 24); not rearrested: 0.00% (0 of 21); overall 42.22% (19 of 45). AUC = 0.396. (D) Model χ^2^(1) = 10.10, *p* = 0.002. Classification of rearrested: 75.00% (18 of 24); not rearrested: 57.14% (12 of 21); overall 66.67% (30 of 45). AUC = 0.661. (E) Model χ^2^(1) = 2.19, *p* = 0.139. Classification of rearrested: 66.67% (16 of 24); not rearrested: 42.86% (9 of 21); overall 55.56% (25 of 45). AUC = 0.548.*

^∗^*p* < 0.05; AUC = area under the curve.

**Table 4:** Cox regression combining ERP or fMRI measures with covariates predicting rearrest (*N* = 45).

(A)								
Predictor	*B*	*Boot-strapped B*	SE *(B)*	*Boot-strapped* SE *(B)*	*p*-value	exp[B]	CI (95%) for exp[B]	*Boot-strapped CI (95%) for exp[B]*
Age at release	-0.05	-0.05	0.04	0.05	0.195	0.95	0.89–1.03	0.86–1.03
PCL-R factor 1	-0.26	-0.26	0.11	0.14	0.022^∗^	0.77	0.62–0.96	0.54–0.93
PCL-R factor 2	-1.37	-1.37	0.46	0.56	0.003^∗^	0.25	0.10–0.62	0.05–0.49
Drug	0.20	0.20	0.24	0.27	0.404	1.22	0.76–1.96	0.76–2.24
Alcohol	-0.31	-0.31	0.29	0.40	0.281	0.73	0.41–1.29	0.29–1.46
ERN/Ne	0.01	0.01	0.06	0.09	0.931	1.01	0.89–1.14	0.86–1.21
Pe	0.09	0.09	0.04	0.06	0.024^∗^	1.10	1.01–1.19	1.02–1.27

*Results of Cox regression analyses examining the predictive effect of the activation accounted for ERP component measures and other covariates on rearrest.*								

**(B)**								
**Predictor**	***B***	***Boot-strapped B***	**SE *(B)***	***Boot-strapped* SE *(B)***	***p*-value**	**exp[B]**	**CI (95%) for exp[B]**	***Boot-strapped CI (95%) for exp[B]***

Age at release	-0.05	-0.05	0.04	0.05	0.137	0.95	0.88–1.02	0.85–1.02
PCL-R factor 1	-0.27	-0.27	0.11	0.14	0.013^∗^	0.76	0.62–0.95	0.53–0.91
PCL-R factor 2	-1.58	-1.58	0.49	0.63	0.001^∗^	0.20	0.08–0.54	0.03–0.39
Drug	0.10	0.10	0.24	0.28	0.683	1.10	0.69–1.75	0.64–1.99
Alcohol	-0.34	-0.34	0.28	0.40	0.230	0.71	0.41–1.24	0.28–1.36
PC1	-0.85	-0.85	1.50	1.96	0.571	0.43	0.02–8.07	0.01–10.98
PC4	2.29	2.29	0.80	1.29	0.004^∗^	9.84	2.04–47.48	2.17–334.95

*Results of Cox regression analyses examining the predictive effect of the activation accounted for principal components derived from ERP measures and other covariates on rearrest.*								

**(C)**								
**Predictor**	***B***	***Boot-strapped B***	**SE *(B)***	***Boot-strapped* SE *(B)***	***p*-value**	**exp[B]**	**CI (95%) for exp[B]**	***Boot-strapped CI (95%) for exp[B]***

Age at release	-0.07	-0.07	0.04	0.04	0.034^∗^	0.93	0.87–0.99	0.83–0.99
PCL-R factor 1	-0.16	-0.16	0.11	0.13	0.147	0.85	0.69–1.06	0.63–1.04
PCL-R factor 2	-0.93	-1.93	0.45	0.50	0.039^∗^	0.39	0.16–0.95	0.11–0.80
Drug	0.20	0.20	0.21	0.26	0.352	1.22	0.80–1.86	0.76–2.14
Alcohol	-0.14	-0.14	0.30	0.40	0.633	0.87	0.49–1.55	0.38–1.89
ACC	-0.53	-0.53	0.29	0.41	0.066^∧^	0.59	0.34–1.04	0.22–1.12

*Results of Cox regression analyses examining the predictive effect of the activation accounted for ACC activation and other covariates on rearrest.*

*^∧^*p* < 0.10; ^∗^*p* < 0.05.*

**Table 5:** Cox regression with ERP, fMRI, and covariates predicting rearrest (*N* = 45).

(A)								
Predictor	*B*	*Boot-strapped B*	SE *(B)*	*Boot-strapped* SE *(B)*	*p*-value	exp[B]	CI (95%) for exp[B]	*Boot-strapped CI (95%) for exp[B]*
Age at release	-0.06	-0.06	0.04	0.05	0.118	0.94	0.87–1.02	0.82–1.01
PCL-R factor 1	-0.21	-0.21	0.12	0.15	0.080^∧^	0.85	0.63–0.1.03	0.55–1.01
PCL-R factor 2	-1.23	-1.23	0.48	0.60	0.011^∗^	0.29	0.11–0.75	0.06–0.58
Drug	0.18	0.18	0.25	0.29	0.440	1.20	0.76–1.91	0.70–1.82
Alcohol	-0.22	-0.22	0.31	0.45	0.484	0.81	0.44–1.48	0.31–1.92
ERN/Ne	0.01	0.01	0.07	0.09	0.860	1.01	0.89–1.15	0.86–1.23
Pe	0.08	0.08	0.04	0.06	0.063^∧^	1.08	1.00–1.18	0.99–1.26
ACC	-0.30	-0.30	0.31	0.46	0.334	0.74	0.40–1.37	0.26–1.58

*Results of Cox regression analyses examining the predictive effect of the activation accounted for ERP component measures, ACC activation, and other covariates on rearrest.*								

**(B)**								
**Predictor**	***B***	***Boot-strapped B***	**SE *(B)***	***Boot-strapped* SE *(B)***	***p*-value**	**exp[B]**	**CI (95%) for exp[B]**	***Boot-strapped CI (95%) for exp[B]***

Age at release	-0.06	-0.06	0.04	0.05	0.137	0.94	0.87–1.02	0.84–1.02
PCL-R factor 1	-0.25	-0.25	0.12	0.16	0.013^∗^	0.78	0.61–0.98	0.51–0.96
PCL-R factor 2	-1.53	-1.53	0.52	0.70	0.001^∗^	0.22	0.08–0.60	0.03–0.44
Drug	0.10	0.10	0.22	0.30	0.683	1.10	0.70–1.74	0.64–2.04
Alcohol	-0.30	-0.30	0.31	0.45	0.230	0.74	0.40–1.36	0.27–1.57
PC1	-1.00	-1.00	1.57	2.08	0.571	0.37	0.02–7.99	0.003–10.54
PC4	2.10	2.10	0.98	1.42	0.004^∗^	8.13	1.19–55.36	1.32–336.17
ACC	-0.12	-0.12	0.37	0.51	0.066^∧^	0.89	0.43–1.83	0.31–2.33

*Results of Cox regression analyses examining the predictive effect of the activation accounted for principal components derived from ERP measures, ACC activation, and other covariates on rearrest.*								

**(C)**								
**Predictor**	***B***	***Boot-strapped B***	**SE *(B)***	***Boot-strapped* SE *(B)***	***p*-value**	**exp[B]**	**CI (95%) for exp[B]**	***Boot-strapped CI (95%) for exp[B]***

ERN/Ne	-0.01	0.01	0.05	0.06	0.843	0.99	0.89–1.10	0.87–1.13
Pe	0.68	0.68	0.03	0.04	0.031^∗^	1.08	1.01–1.14	1.00–1.17
ACC	-0.27	-0.27	0.21	0.27	0.200	0.76	0.50–1.16	0.43–1.25

*Results of Cox regression analyses examining the predictive effect of the activation accounted for ERP component measures and ACC activation on rearrest.*								

**(D)**								
**Predictor**	***B***	***Boot-strapped B***	**SE *(B)***	***Boot-strapped* SE *(B)***	***p*-value**	**exp[B]**	**CI (95%) for exp[B]**	***Boot-strapped CI (95%) for exp[B]***

PC1	-0.63	-0.63	1.17	1.39	0.591	0.53	0.05–5.28	0.03–7.48
PC4	1.46	1.46	0.70	0.91	0.039^∗^	4.29	1.08–17.04	0.91–32.30
ACC	-0.18	-0.18	0.23	0.27	0.421	0.83	0.54–1.30	0.47–1.40

*Results of Cox regression analyses examining the predictive effect of the activation accounted for principal components derived from ERP measures and ACC activation on rearrest.*

*^∧^*p* < 0.10; ^∗^*p* < 0.05.*

Logistic regressions were computed to identify variables useful in predicting who will or will not be rearrested in a linear analysis. Similar to the Cox regressions presented above, the neural measures of error-monitoring and post-error processing were included in models with the covariates (**Tables 6** and **7**). Again, the principal component measure of post-error processing was most sensitive to identifying who would or would not be rearrested. The best logistic regression model included PCA and ACC activation measures. In this model, individuals were identified at 65.85% rate overall with 65.00% correctly identified as not being rearrested and 66.67% correctly identified as rearrested (AUC = 0.658; **Table 7D**). PC4, *p* = 0.038 was the only unique predictor in this model. For every unit increase in PC4 amplitude, there was a 38.31 increase in the probability of rearrest. Generalizability of these logistic regression models is enhanced because of the out of sample cross-validation implemented here.

**Table 6:** Logistic regressions combining ERP and fMRI with covariates predicting rearrest (*N* = 45).

(A)					
Predictor	*B*	SE *(B)*	*p*-value	exp[B]	CI (95%) for exp[B]
Age at release	-0.02	0.06	0.762	0.98	0.88–1.10
PCL-R factor 1	-0.24	0.16	0.147	0.79	0.57–1.08
PCL-R factor 2	-1.67	0.84	0.048^∗^	0.19	0.04–0.98
Drug	0.07	0.37	0.819	1.08	0.52–2.25
Alcohol	-0.48	0.53	0.373	0.62	0.22–1.76
ERN/Ne	-0.0006	0.10	0.895	1.00	0.82–1.22
Pe	0.17	0.09	0.050^∗^	1.18	1.00–1.40

*Results of logistic regression analyses examining the predictive effect of the activation accounted for in TD ERP components, and other covariates on rearrest. -2 Log likelihood = 41.48 (SD = 1.28), Cox and Snell *R^*2*^* = 0.28 (SD = 0.009); Nagelkerke *R^*2*^* = 0.38 (SD = 0.012); Model χ^*2*^(7) = 13.69, *p* = 0.058. Classification of rearrested: 66.67% (14 of 21); not rearrested: 55.00% (11 of 20); overall 60.98% (25 of 41). AUC = 0.608.*					

**(B)**					
**Predictor**	***B***	**SE *(B)***	***p*-value**	**exp[B]**	**CI (95%) for exp[B]**

Age at release	-0.03	0.06	0.600	0.97	0.86–1.09
PCL-R factor 1	-0.24	0.18	0.165	0.78	0.56–1.10
PCL-R factor 2	-2.26	0.97	0.020^∗^	0.11	0.02–0.69
Drug	0.07	0.39	0.834	1.08	0.50–2.35
Alcohol	-0.71	0.59	0.235	0.50	0.16–1.56
PC1	-0.26	2.49	0.877	1.57	0.01–339.98
PC4	6.23	2.38	0.009^∗^	1712.50	5.28–2015200

*Results of logistic regression analyses examining the predictive effect of the activation accounted for in ERP principal components and other covariates on rearrest. -2 Log likelihood = 35.53 (SD = 1.76), Cox and Snell *R^*2*^* = 0.38 (SD = 0.014); Nagelkerke *R^*2*^* = 0.51 (SD = 0.019); Model χ^2^(7) = 19.68, *p* = 0.007. Classification of rearrested: 66.67% (14 of 21); not rearrested: 60.00% (12 of 20); overall 63.41% (26 of 41). AUC = 0.633.*					

**(C)**					
**Predictor**	***B***	**SE *(B)***	***p*-value**	**exp[B]**	**CI (95%) for exp[B]**

Age at release	-0.09	0.05	0.105	0.92	0.82–1.02
PCL-R factor 1	-0.06	0.16	0.706	0.94	0.69–1.28
PCL-R factor 2	-0.91	0.64	0.162	0.40	0.12–1.42
Drug	0.02	0.36	0.886	1.02	0.50–2.08
Alcohol	-0.07	0.51	0.851	0.94	0.35–2.54
ACC	-0.98	0.58	0.093^∧^	0.38	0.12–1.17

*Results of logistic regression analyses examining the predictive effect of the activation accounted for in the ACC and other covariates on rearrest. -2 Log likelihood = 44.91 (SD = 1.24), Cox and Snell *R^*2*^* = 0.22 (SD = 0.008); Nagelkerke *R^*2*^* = 0.30 (SD = 010); Model χ^2^(6) = 10.26, *p* = 0.115. Classification of rearrested: 61.90% (13 of 21); not rearrested: 55.00% (11 of 20); overall 58.54% (24 of 41). AUC = 0.585.*

*^∧^*p* < 0.10; ^∗^*p* < 0.05; AUC = area under the curve.*

**Table 7:** Logistic regressions combining ERP, fMRI, and covariates predicting rearrest (*N* = 45).

(A)					
Predictor	*B*	SE *(B)*	*p*-value	exp[B]	CI (95%) for exp[B]
Age at release	-0.04	0.06	0.565	0.97	0.85–1.09
PCL-R factor 1	-0.16	0.17	0.376	0.85	0.61–1.20
PCL-R factor 2	-1.58	0.86	0.069^∧^	0.21	0.04–1.11
Drug	-0.02	0.40	0.882	0.99	0.45–2.17
Alcohol	-0.28	0.57	0.613	0.76	0.25–2.36
ERN/Ne	-0.02	0.10	0.841	0.98	0.80–1.21
Pe	0.16	0.09	0.076^∧^	1.18	0.99–1.41
ACC	-0.75	0.62	0.235	0.48	0.14–1.60

*Results of logistic regression analyses examining the predictive effect of the activation accounted for in TD ERP components, the ACC, and other covariates on rearrest. -2 Log likelihood = 39.80 (SD = 1.46), Cox and Snell *R^*2*^* = 0.31 (SD = 0.013); Nagelkerke *R^*2*^* = 0.41 (SD = 0.018); Model χ^2^(8) = 15.23, *p* = 0.057. Classification of rearrested: 61.90% (13 of 21); not rearrested: 60.00% (12 of 20); overall 60.98% (25 of 41). AUC = 0.610.*					

**(B)**					
**Predictor**	***B***	**SE *(B)***	***p*-value**	**exp[B]**	**CI (95%) for exp[B]**

Age at release	-0.05	0.06	0.495	0.96	0.84–1.09
PCL-R factor 1	-0.18	0.20	0.366	0.84	0.57–1.23
PCL-R factor 2	-2.17	0.99	0.028^∗^	0.12	0.02–0.79
Drug	0.01	0.41	0.902	1.01	0.45–2.28
Alcohol	-0.57	0.61	0.357	0.57	0.17–1.87
PC1	0.09	2.58	0.905	1.34	0.01–369.55
PC4	5.97	2.43	0.015^∗^	1625	3.77–2668700
ACC	-0.53	0.74	0.478	0.59	0.15–2.50

*Results of logistic regression analyses examining the predictive effect of the activation accounted for in ERP principal components, the ACC, and other covariates on rearrest. -2 Log likelihood = 34.96 (SD = 1.82), Cox and Snell *R^*2*^* = 0.39 (SD = 0.16); Nagelkerke *R^*2*^* = 0.52 (SD = 0.021); Model χ^2^(8) = 20.17, *p* = 0.011. Classification of rearrested: 61.90% (13 of 21); not rearrested: 60.00% (12 of 20); overall 60.98% (25 of 41). AUC = 0.610.*					

**(C)**					
**Predictor**	***B***	**SE *(B)***	***p*-value**	**exp[B]**	**CI (95%) for exp[B]**

ERN/Ne	0.008	0.09	0.880	1.01	0.84–1.21
Pe	0.13	0.07	0.055^∧^	1.14	1.00–1.30
ACC	-0.61	0.45	0.183	0.54	0.23–1.32

*Results of logistic regression analyses examining the predictive effect of the activation accounted for in TD ERP components and the ACC on rearrest. -2 Log likelihood = 47.24 (SD = 0.98), Cox and Snell *R^*2*^* = 0.18 (SD = 0.005); Nagelkerke *R^*2*^* = 0.24 (SD = 0.007); Model χ^2^(3) = 8.13, *p* = 0.044. Classification of rearrested: 61.90% (13 of 21); not rearrested: 60.00% (12 of 20); overall 60.98% (25 of 41). AUC = 0.610.*					

**(D)**					
**Predictor**	***B***	**SE *(B)***	***p*-value**	**exp[B]**	**CI (95%) for exp[B]**

PC1	-0.22	1.96	0.866	0.98	0.02–78.58
PC4	2.40	1.62	0.038^∗^	38.31	1.32–1436.42
ACC	-0.47	0.45	0.306	0.63	0.26–1.52

*Results of logistic regression analyses examining the predictive effect of the activation accounted for in ERP principal components and the ACC on rearrest. -2 Log likelihood = 46.62 (SD = 1.22), Cox and Snell *R^*2*^* = 0.19 (SD = 0.01); Nagelkerke *R^*2*^* = 0.25 (SD = 0.01); Model χ^2^(3) = 8.67, *p* = 0.035. Classification of rearrested: 66.67% (14 of 21); not rearrested: 65.00% (13 of 20); overall 65.85% (27 of 41). AUC = 0.658.*

*^∧^*p* < 0.10; ^∗^*p* < 0.05; AUC = area under the curve.*

To evaluate non-linear combinations of these variables predicting rearrest, SVM models were computed. Four simple models were computed containing: (1) only the covariates, (2) only the TD measures (ERN/Ne, and Pe), (3) only the PCA measures (PC1 and PC4), (4) or only ACC activity. Additional models were computed which combined ERP TD, ERP PCA, ACC activation, and covariate measures (**Table 8**). Several of these models successfully predicted either who will be rearrested or who will not be rearrested. The model that predicted both who will and will not be rearrested included the ERP PCA and ACC activation measures (**Table 8B**). This model had an overall accuracy of 78.05% while accurately predicting who will (83.33%) and will not (70.59%) be rearrested. Similar to the logistic regression models, the SVM models presented here were cross-validated thereby increasing the generalizability of these results.

**Table 8:** Support vector machine analyses with ERP, fMRI, and covariates predicting rearrest.

(A)					
	Covariates	Time-domain measures	PCA measures	Covariates with TD Measures	Covariates with PCA measures
Overall classification rate	60.98%	65.85%	70.73%	73.17%	70.73%
Specificity	70.83%	95.83%	58.33%	83.33%	66.67%
Sensitivity	47.06%	23.53%	88.24%	58.82%	76.47%
Positive predictive value	53.33%	80.00%	60.00%	71.43%	61.90%
Negative predictive value	65.38%	63.89%	87.50%	74.07%	80.00%

*Five support vector machine (SVM) models predicting rearrest were computed individually for covariates (age at release, PCL-R factor 1, PCL-R factor 2, drug dependencies, and alcohol dependencies), component windowed TD ERP measures (mean of ERN/Ne and Pe), and principal component (PCA) ERP measures (mean of PC1 and PC4). Covariates and ERP measures (either TD or PCA measures) were included in additional SVM models. Specificity is the measure of how well the model identified who will be rearrested and sensitivity is the measure of how well the model identified who will not be rearrested. Positive predictive value represents the ratio of individuals who were not rearrested to combined individuals identified correctly and incorrectly to not be rearrested. Negative predictive value represents the ratio of individuals who were rearrested to combined individuals identified correctly and incorrectly to be rearrested.*					

**(B)**						
	**ACC**	**ACC with covariates**	**ACC with TD measures**	**ACC with PCA measures**	**ACC, TD, and covariates**	**ACC, PCA, and covariates**

Overall classification rate	68.29%	63.41%	70.73%	78.05%	68.29%	73.17%
Specificity	62.50%	58.33%	66.67%	83.33%	66.67%	66.67%
Sensitivity	76.47%	70.59%	76.47%	70.59%	70.59%	82.35%
Positive predictive value	59.09%	54.55%	61.90%	75.00%	60.00%	63.64%
Negative predictive value	78.95%	73.68%	80.00%	80.00%	76.19%	84.21%

*Six SVM models predicting rearrest were computed for the hemodynamic activation measured in the anterior cingulate cortex (ACC) along with covariates (age at release, PCL-R factor 1, PCL-R factor 2, drug dependencies, and alcohol dependencies), and ERP measures (component windowed TD and PCA measures). Specificity is the measure of how well the model identified who will be rearrested and sensitivity is the measure of how well the model identified who will not be rearrested. Positive predictive value represents the ratio of individuals who were not rearrested to combined individuals identified correctly and incorrectly to not be rearrested. Negative predictive value represents the ratio of individuals who were rearrested to combined individuals identified correctly and incorrectly to be rearrested.*

Each of the three analytical approaches (Cox regression, logistic regression, and SVM) was successful in classifying either time to rearrest or whether someone will or will not be rearrested. Arguably the strongest models with out of sample cross-validation presented here with this sample included multimodal neural measures of error-processing. In all three analytical approaches, the models that included the PCA measure of ERPs and ACC activation were most useful in prospectively predicting time to rearrest and classifying individuals who will and will not be rearrested. The strongest unique predictor in these models proved to be PC4 which corresponds to the Pe. This ERP component, as discussed below, is interpreted as a measure of post-error processing. However, in the SVM models, the combination of ERP and fMRI measures without other covariates was most successful in classifying groups suggesting multimodal imaging measures are well suited to these types of prediction models.

## Discussion

The present study marks a first attempt at prospective neuroprediction of rearrest by measuring post-error processing in both ERPs and functional magnetic resonance imaging (fMRI). Multimodal and individual models including ERP and fMRI measures accurately classified individuals who would or would not be rearrested. SVM models which included ERP and fMRI measures proved to be most sensitive in predicting outcomes compared to Cox and logistic regressions. The best SVM model was 78.05% accurate overall while identifying 83.33% of those who were rearrested and 70.59% of those who were not rearrested. This model included PCA of ERPs and ACC activation elicited by response errors. Neural measures predicted outcomes over other, traditional measures (i.e., the covariates in these models). Age of release, PCL-R Factor 1, and PCL-R Factor 2 were also identified as predictors of rearrest although the best prediction models only included the neural measures of post-error processing. A multimodal neuroimaging approach isolating post-error processes is highlighted here allowed for reliable prospective predictions of antisocial behavior (i.e., rearrest).

Increased Pe amplitude and reduced ACC hemodynamic activity measured here prospectively predicted rearrest. The Pe, measured in response inhibition tasks, has been known to index further error-processing, conscious evaluation of the error, response strategy adjustments, and/or affective assessment of the error (Falkenstein et al., 1991; Nieuwenhuis et al., 2001; Overbeek et al., 2005). The Pe has been linked to ACC activation (van Veen and Carter, 2002; Edwards et al., 2012) and to poor future outcomes in drug treatment (Steele et al., 2014b). More specifically, increased Pe, as identified here, has been linked to decreased ACC activation (Edwards et al., 2012) which is in-line with our previous report that low ACC activation predicts rearrest (Aharoni et al., 2013).

With sophisticated analytical techniques used here (SVM), it was possible to accurately predict rearrest from increased Pe amplitude and decreased hemodynamic activation in the ACC. In the current sample, ERN/Ne did not predict outcomes. Identifying the Pe and not the ERN/Ne as predictive of rearrest isolates post-error processing as the cognitive function associated with poor future outcomes. Individuals who differentially process errors may have difficulty learning from mistakes which may lead to poor behavioral outcomes. More specifically, offenders with abnormal post-error processes may be at greater antisocial risk because response errors are not categorized as events to be minimized in the future. Understanding which specific cognitive function is important in that neural responses are relatively dynamic and amenable to change (Greely, 2009; Woltering et al., 2011; Larson et al., 2013) thus holding an advantage over other risk factors (e.g., age of release). Treatment specifically targeting conscious awareness of errors and modifying behavior to avoid future errors may be useful in future interventions to remediate antisocial (or impulsive) behavior and reducing Pe amplitudes (c.f. Larson et al., 2013).

Although the present findings extend previous neuroprediction models, caution should be exercised when applying these findings to real-world situations. Incremental validity in neural measures of post-error processing was demonstrated over and above actuarial measures in predicting rearrest and time to rearrest. However, our sample was limited to 45 participants and the fMRI analysis was limited to activation within the ACC. Both Age and ACC activation were less predictive of future outcomes compared to previous reports which included 96 participants (Aharoni et al., 2013) most likely because of the reduced sample size. Replications and larger samples are necessary to isolate any specific indicator variables between neural measures and future behavior. Additional fMRI analysis including other regions of interest identified in this task (Steele et al., 2013, 2014a) or more sophisticated analysis techniques (e.g., ICA) could be used to identify networks that are predictive of rearrest. Moreover, we highlight here the need for replications and extension of these findings. Specifically, such analyses should be carried out in youth samples to help identify prediction measures that could be targeted prior to reaching adulthood. Also, the current sample only included incarcerated males. Future samples should use females and parolees to extend the current findings. Finally, a direct comparison of neural measures of error-processing between incarcerated individuals who did or did not reoffend and healthy controls is necessary to fully delineate specific cognitive functions or dysfunctions related to risk of reoffending. With such a comparison, stronger conclusions could be made toward the Pe and ACC activation differences highlighted here and the relation to a healthy control sample. Finally, the heterogeneity of reoffenses in the current sample allowed for little interpretation about risk-factors related to specific types of offense. Future studies could employ a larger sample of homogeneous offenders that could help with interpretation of specific offense outcomes. Each of these analyses, paired with the current findings, could lead to the development of targeted interventions and treatments for those individuals at greatest risk of rearrest. Allocating scarce resources to individuals with the greatest need could potentially reduce incidents of future arrest thus reducing the societal cost of crime overall. Finally, we caution the use of our findings, and any neuropredictive measures at this point, to identify any single individual’s risk. Presented here are group-level analyses to be only interpreted in that context.

These results extend recent findings by Aharoni et al. (2013), previous accounts that increased ACC activation is associated with improved inhibitory control (Kerns et al., 2004), and increased Pe amplitude to be related to poor future outcomes (Steele et al., 2014b). Both ERP and fMRI measures of error-processing prospectively predict rearrest. SVM models provided the strongest models relative to Cox and logistic regressions though each of these models was successful in prospectively predicting outcomes. Individuals with increased Pe amplitude and decreased ACC activation, specifically indexing error-processing, were more likely to be rearrested after release from incarceration. These findings should be used to develop new treatments specifically targeting conscious processing of response errors in an attempt to reduce future rearrests. Recent mindfulness treatments have specifically targeted such processes while reducing Pe amplitudes (Larson et al., 2013) and should be seriously considered as an intervention in individuals at greatest risk for poor future outcomes. The power of multimodal predicting is highlighted here in the sensitivity demonstrated within this relatively small sample. This power is yet to be fully realized and future explorations of this topic, with larger samples, are necessary to solidify the findings reported here.

## Author Contributions

KK and VC developed the study design. VS performed the data analysis and interpretation with contributions by EA, EC, and GV. VS drafted the manuscript and all authors provided critical revisions. All authors approved the final version for submission.

## Conflict of Interest Statement

The authors declare that the research was conducted in the absence of any commercial or financial relationships that could be construed as a potential conflict of interest.
